# Sometimes the impact factor outshines the H index

**DOI:** 10.1186/1742-4690-5-88

**Published:** 2008-10-06

**Authors:** Johannes Hönekopp, Janet Kleber

**Affiliations:** 1 Northumbria University, Department of Psychology, Ellison Square, Newcastle upon Tyne, NE1 8ST, UK; 2 Universität Erfurt, Erziehungswissenschaftliche Fakultät, Fachgebiet Psychologie, Nordhäuserstr. 63, 99089 Erfurt, Germany

## Abstract

Journal impact factor (which reflects a particular journal's quality) and H index (which reflects the number and quality of an author's publications) are two measures of research quality. It has been argued that the H index outperforms the impact factor for evaluation purposes. Using articles first-authored or last-authored by board members of Retrovirology, we show here that the reverse is true when the future success of an article is to be predicted. The H index proved unsuitable for this specific task because, surprisingly, an article's odds of becoming a 'hit' appear independent of the pre-eminence of its author. We discuss implications for the peer-review process.

## Introduction

Recently, Jeang [1] argued forcefully for the use of individualized citation metrics instead of measures of journal quality for evaluation purposes. Before the age of personal computers, so Jeang argues, judging an article by the quality of the journal was almost inevitable; but as individualized citation statistics have become readily available, it appears outdated to "judge a book by its cover". We agree with Jeang that individual merit is suitably measured by individualized citation metrics, which also predict scientists' future success well [2]. But we also contend that "judging a book by its cover" (i) is deeply engrained in human nature [3], (ii) can be adaptive because outward appearance is often a probabilistic cue to some hidden quality [4,5], (iii) and is often without alternative. Imagine you want to decide which new articles to read outside your narrow field of specialization. How can you decide which ones are worthy of your time when citation frequencies are not yet available? You may infer article quality from an individualized citation metric like the H index of the author (with H being the largest number of publications of an author that have been cited at least H times); alternatively, you may base your inference on a measure of journal quality like its impact factor (IF, which reflects the average citation frequency of articles from a particular journal).

Previous research suggests that the IF may outperform the H index in predicting an article's number of citations, which is often used as a proxy for article quality [2,6,7]. Not because IFs work particularly well – as Jeang [1] correctly noted, citation frequencies vary greatly for articles in the same journal – but because the H index should be completely unsuitable for this specific task. This is because authors who publish the most highly cited publications also publish the highest number of ignored publications [6]. As a consequence, a counter-intuitive *equal-odds rule *[7] is at work, whereby an article's probability of becoming a great success is independent of the number of articles of its author. Therefore, the number of citations of an article should be independent of the pre-eminence (and thus, of the H index) of its author.

## IF and H index as predictors of article citation frequencies

In order to test this prediction, we investigated to what extent the citation frequency of an article can be predicted from the H index of the first author and the journal's IF. Following Jeang [1], we concentrated on the 45 editorial board members of Retrovirology as of June 2007. Using Google Scholar, we searched for their publications as first authors between 2002 and 2005. Unambiguous information about authorship and IF was available for 97 articles by 29 board members. We used IFs from 2006 throughout because this was the earliest year for which all relevant IFs could be obtained. We used authors' H indexes from the respective year of publication, which are easy to research [8].

**Figure 1:**
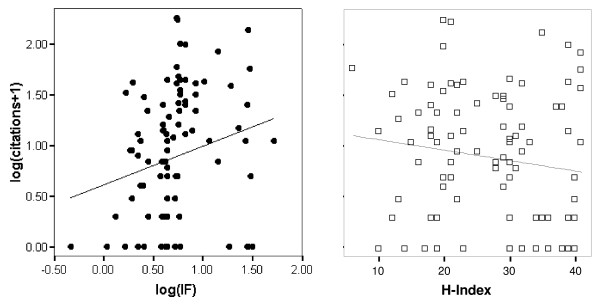
Article citation frequency is predicted by journal impact factor (*r *= .22, *p *= .029) but not by first author's H-index (*r *= -.15, *p *= .16).

IFs and article citation frequencies were heavily right skewed and therefore log-transformed. To predict log(citations+1), we fed first-authors' H index and log(IF) into a stepwise linear regression. As citation frequency should be negatively related to publication year, we also included the latter as a predictor. A significant model resulted (*R *= .33, *F*_2,94 _= 5.7, *p *= .005), with the regression equation log(citations+1) = 308.05 – 0.15 publication year + 0.40 log(IF). Log(IF) proved to be a significant predictor (β = .23, *t*_94 _= 2.36, *p *= .021); the same held true for publication year (β = -.24, *t*_94 _= 2.48, *p *= .015). Interestingly, and in line with our prediction, H index did not predict log(citations+1) (β = -.13, *t*_94 _= 1.34, *p *= .19). The first order correlation between log(IF) and log(citations+1) was significant and positive (*r *= .22, *p *= .029) and is depicted in Figure 1. As expected, the first order correlation between H index and log(citations+1), which is also depicted in Figure 1, was not significant and even slightly negative (*r *= -.15, *p *= .16).

Most board members of Retrovirology may have reached a stage in their career in which those papers are most representative of their work for which they are last author. We therefore repeated the above analysis with papers on which board members were last author; 324 relevant papers were obtained.

A stepwise regression analysis resulted in a significant model (*R *= .31, *F*_2,321 _= 16.8, *p *< .001), with log(citations+1) = 259.07 – 0.13 publication year + 0.26 log(IF). Log(IF) proved to be a significant predictor (β = .17, *t*_321 _= 3.13, *p *= .002); the same held true for publication year (β = -.27, *t*_321 _= 5.11, *p *< .001). As hypothesized, last author's H index did not predict log(citations+1) (β = -.07, *t*_321 _= 1.27, *p *= .20). The first order correlation between log(IF) and log(citations+1) was significant and positive (*r *= .15, *p *= .009). As expected, the first order correlation between last author's H index and log(citations+1) was not significant and again even slightly negative (*r *= -.06, *p *= .26).

## Conclusion

A journal's IF reflects how often, on average, articles in this journal are cited. Therefore, the IF must be able to predict an article's future citations, which are often seen as a proxy for its quality [2,6,7]. In two samples of articles first-authored or last-authored by the board members of Retrovirology, we found that the predictive power of the IF was surprisingly small, which may be an effect of the low reliability of the peer review system [9]. However, previous research on creativity [6,7] suggests that an author's H index, which is very successful at predicting scientists' future success [2], should fail to predict an article's future citations. Our results fully confirm this counter intuitive prediction. As our finding is in line with previous findings on creativity [6,7], we are confident that it can be replicated with other, less specific samples. Our findings thus suggest that for the specific task of prediction the future citations of an article the IF outshines the H index. Consequently, when deciding which new articles to read outside their field of specialization, readers are likely to find some guidance in the prestige of journals but none in the prestige of authors.

We believe that our findings have important implications for the peer-review process. Reviewers are biased in favour of prestigious authors [9]. This appears highly undesirable given that authors' pre-eminence (as measured by the H index) appears unable to predict article quality. Knowledge about cognitive biases is often not sufficient to overcome them [10]. Therefore, it might be difficult for reviewers to immunize themselves against this "prestigious-authors bias" even if they are aware of it. Deleting authors' names and affiliations from reviewed manuscripts appears a viable alternative. Experienced reviewers may correctly feel that they can often guess a submission's author even if this information is omitted. For two reasons, this is not an argument against blind reviewing. First, guessing correctly is not the same as guessing well, as a simple example shows. Assume that 70% of the manuscripts a particular reviewer receives originate from lab A. Further assume that the reviewer correctly attributes 80% of these submissions to lab A, but that the reviewer also attributes 80% of the other submissions to lab A (after all, these submissions are likely to cite many publications from lab A, use similar techniques, etc.). In this case, the reviewer often guesses correctly but is unable to discriminate between lab A and other labs. Second and more importantly, omitting author information from submissions does not require much effort. Therefore, the benefit of blind reviewing will sufficiently outweigh its costs even if it works only at times.

## Competing interests

The authors declare that they have no competing interests.

## Authors' contributions

The authors collaborated closely on all aspects of the work.
